# Decreasing cholesterol levels in the community – lifestyle change with statin?

**DOI:** 10.1186/s12875-015-0240-y

**Published:** 2015-02-28

**Authors:** Jorma Savolainen, Hannu Kautiainen, Leo Niskanen, Pekka Mäntyselkä

**Affiliations:** Institute of Public Health and Clinical Nutrition, Primary Health Care, School of Medicine, University of Eastern Finland, P.O. Box 1627, FI-70211 Kuopio, Finland; Primary Health Care Unit, Kuopio University Hospital, P.O. Box 100, FI-72009 KYS Kuopio, Finland; Department of General Practice, Helsinki University Central Hospital, Unit of Primary Health Care and University of Helsinki, P.O. Box 20, FI-00014 Helsinki, Finland; Endocrinology, Abdominal Center, Helsinki University Central Hospital, P.O. Box 55, FI- 00014 Helsinki, Finland

**Keywords:** Cholesterol, Lifestyle, Population-based study

## Abstract

**Background:**

The Lapinlahti 2005–2010 study was carried out to explore cardiovascular disease risk factors and changes in lifestyle in Lapinlahti residents in eastern Finland. Our aim was to analyse factors influencing the level of cholesterol in the community.

**Methods:**

In 2005, 480 subjects aged 30–65 years underwent a complete health survey (baseline study) that consisted of a structured questionnaire and a health examination. The follow-up was carried out five years later in 2010. The present study population included 326 individuals who did not use lipid-lowering medication at the baseline. A trained research nurse measured weight, height, waist circumference and blood pressure at the baseline and follow-up. Respectively, lifestyle factors (nutrition, exercise, smoking and alcohol use) were examined with a structured questionnaire. Each lifestyle item was valued as −1, 0 or 1, depending on how closely it fitted to the recommendations. Cholesterol level analyses at the baseline and follow-up were performed according to the protocol of the Kuopio University Hospital’s medical laboratory. Based on their baseline cholesterol levels, the participants were divided into tertiles. The age- and sex-adjusted linear trend between the tertiles was tested.

**Results:**

The change in cholesterol level was associated with lipid-lowering medication (P < 0.001). Lifestyle improvement was associated with the cholesterol level change but did not reach statistical significance (P = 0.061), although the interaction of lipid-lowering medication and lifestyle change was associated with the change in cholesterol level (P = 0.018). In multivariate analysis, a favourable change in fat consumption (P = 0.007) and lipid-lowering medication (P < 0.001) were associated with decreasing cholesterol levels.

**Conclusions:**

At the population level, dyslipidaemia is one of the most easily modifiable risk factors of CHD. Lipid-lowering medication may have the most significant impact on cholesterol level in communities with primary health care with good coverage. On the other hand, the potential of health-promoting and population-based prevention strategies may be underused.

## Background

Cardiovascular diseases, of which coronary heart disease (CHD) is the most common, are the major cause of death in most European countries [1]. Serum cholesterol levels are linearly related to CHD mortality. Furthermore, there is a linear relationship between the increase in CHD mortality and an increase in cholesterol levels [2]. Evidence from clinical trials as well as from epidemiological studies indicates that dyslipidaemia is one of the most important modifiable risk factors for CHD [3,4]. Cholesterol levels are determined by multiple genetic factors as well as by environmental factors—primarily dietary habits [5]. According to current clinical CHD treatment guidelines, prevention should be based on an assessment of each individual’s overall risk of atherosclerotic vascular diseases. If the risk is significantly increased, it should be lowered through lifestyle changes and/or medical treatment [6,7].

Finland has a long tradition of public health policies with many health promotion programmes, of which the North Karelian project (launched in 1972) in eastern Finland is the most well-known worldwide. The work done in Finland has led to greatly improved quantity and quality of dietary fat intake and a remarkable reduction in blood cholesterol levels [8]. Serum cholesterol levels declined markedly in Finland from 1972 to 2007, as did CHD mortality among the middle-aged [9]. However, according to the FINRISKI 2012 study, this decline in serum cholesterol levels has levelled off and actually had increased recently (1.7% in men and 3.1% in women during 2007–2012) [10].

Clinical trials in humans have shown that lowering the level of serum cholesterol with diet or drugs (statins) decreases subsequent incidence of fatal or nonfatal CHD [3]. Many large clinical trials have demonstrated that statins substantially reduce cardiovascular morbidity and mortality in both primary and secondary prevention [6,8,11]. Modification of diet, especially reductions in saturated fat and cholesterol, effectively reduce serum cholesterol concentrations, but there is considerable heterogeneity in individual responses to cholesterol-lowering diets [12]. There is also evidence that statin users seem to adopt a healthier lifestyle than non-statin users. This may be because statin users are likely to be repeatedly exposed to information from health professionals, and at the same time their high-risk lipid profile necessitating regular intake of drugs may have wide-spread favourable reflections on their health, but the precise mechanism behind this association is unknown [11].

Primary care physicians play a key role in identifying and treating dyslipidaemia patients. In each case, lifestyle modification should play an integral role in the management programme with additive pharmacologic therapy, if required [13]. Most articles published in medical literature documenting successes in lipid lowering originate from large academic centres or very large, well-funded studies. The challenge for community-based physicians is to put these lipid-lowering guidelines, standards and strategies into everyday medical practice [14]. The aim of the present study was to analyse the population’s cholesterol level changes during the five-year follow-up period in the primary health care setting.

## Methods

Lapinlahti, with a total population of 10289, is a typical semi-rural municipality in eastern Finland with a demographic shift to older age strata and increasing migration of the young and economically active population to urban centres [15]. All adults born in 1939, −44, −49, −54, −59, −64, −69 and −74 living in Lapinlahti municipality in Eastern Finland were included in the original study sample based on the Finnish National Population registry [16].

In 2005, 480 subjects (63% of the whole study population230 men and 250 women) underwent a complete health survey (baseline study) that consisted of a structured questionnaire and a health examination [17]. After the health check in 2005, all participants were sent a written feedback by the researcher (physician). If necessary, it included advice to e.g. quit smoking, decrease alcohol use and to eat more vegetables, fruits and berries and exercise more.

The present study is based on the five-year follow-up of the baseline cohort (2005–2010) (N = 376, no. of males = 184). Age, sex distribution, educational level, marital status, employment status and BMI in subjects who were lost from the follow-up or did not respond were similar to those who were followed up. The use of continuous medication was enquired at both examinations. Those who had cholesterol-lowering medication in 2005 were excluded from these analyses. Hence, the present study cohort included 326 individuals (no. of males = 158).

**Figure 1:**
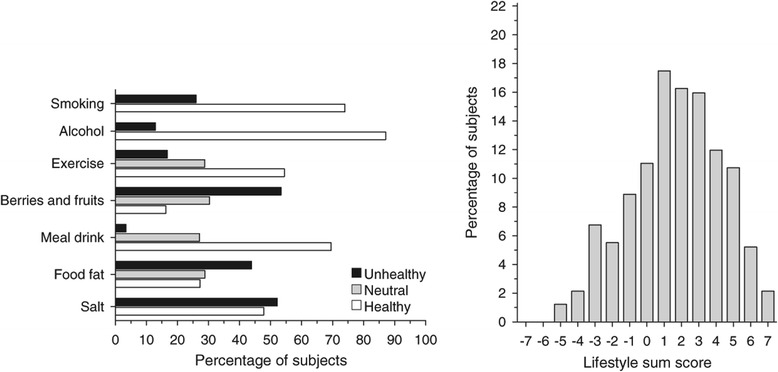
**Distribution of the subjects in the particular lifestyle scores and in the lifestyle sum scores.**

The study participants filled out a structured questionnaire at both the baseline and follow-up. The questionnaire included lifestyle items regarding smoking, alcohol use, exercise and nutrition. Based on the national guidelines and health recommendations [18,19], we ranked lifestyle as follows: meal beverage (+1 = water or non-fat milk, 0 = skimmed milk or sour milk, −1 = fatty milk or something else), cooking fat (+1 = nothing or margarine, dairy spread, 0 = mixture of butter and vegetable oils, −1 = butter or something else), spreads (+1 = nothing or margarine spread, 0 = mixture of butter and vegetable oils, −1 = butter or something else), cooking fat and spreads were combined; use of vegetables (+1 = used more than 6 times/week, 0 = used from 1 to 5 times /week, −1 = never or occasional); berry and fruit intake (+1 = used more than 6 times/week, 0 = from 1 to 5 times /week, −1 = never or occasionally), vegetable, berry and fruit intake (VBF) were also combined into a new variable as was done with fats; adding salt to food (+1 = never, 0 = usually when food doesn’t taste salty enough, −1 = often even without tasting); alcohol consumption (male: +1 = less than 5 doses/week, −1 = over 5 doses/week or more, female: +1 = less than 4 doses/week and −1 = 4 doses/week or more); smoking (+1 = never smoking, −1 = regular or irregular smoking); exercise (+1 = daily or more often, 0 = from 1 to 6 times /week, −1 = less than one time/week). All these values were summed up and a mean value (ranging from −1 to +1) was calculated for each participant. The participants were divided into tertiles (I = unhealthy, II = neutral and III = healthy) according to the value of the sum score [20]. Figure 1 shows the distribution of particular lifestyle scores and the distribution of sum score. In addition, depressive symptoms were assessed using the 21-item Beck Depression Inventory (BDI-21), and a BDI-21 score of 10 was used as a cut-off point for depressive symptoms [21].

The health examination conducted at the baseline and follow-up included measurements of weight and height, blood pressure and waist circumference. Weight was measured in light clothing with a calibrated Camry® standing scale to the nearest 0.1 kg, and height was measured in a standing position to the nearest 1.0 cm. Body mass index (BMI) was calculated as weight/height^2^. Waist circumference was measured at the midpoint between the lowest rib and the iliac crest. For the blood pressure measurements, we used an Omron® M3 semiautomatic device. Blood pressure was taken in a sitting position at five-minute intervals after 10 minutes of rest. For the statistical analysis, we calculated the means of the three measurements.

All the laboratory investigations were performed according to the protocol of the Kuopio University Hospital’s medical laboratory and the values are based on the fasting samples. Glucose level was tested from capillary blood with a glucometer (HemoCue 201) calibrated for plasma glucose level. Other laboratory tests were done from the serum of venous blood. Lipid levels were measured with a Cobas 6000 analyser as follows: plasma cholesterol by using a colorimetric enzymatic assay, plasma high-density lipoprotein (HDL) cholesterol by using a homogenous enzymatic colorimetric assay, plasma low-density lipoprotein (LDL) cholesterol by using a homogenous enzymatic colorimetric assay and triglycerides by using an enzymatic colorimetric test.

The data are presented as means and standard deviations or as counts with percentages. Statistical comparisons were made by using the chi-square test or analysis of variance (ANOVA). In the case of violation of the assumptions (e.g., on-normality), a bootstrap-type test was used. The bootstrap method is significantly helpful when the theoretical distribution of the test statistic is unknown or in case of violation of the assumptions. To determine characteristics associated with change in serum cholesterol levels, univariate and forward stepwise multivariate regression analysis was applied. No adjustment was made for multiple testing. The STATA 13.1 (StataCorp LP, College Station, TX, USA) statistical package was used for the analyses.

All the participants signed an informed consent form. This study was carried out in compliance with the Helsinki Declaration. Ethical permission for the study was granted by the ethics committee of the Kuopio University Hospital (number 129/2009).

## Results

The demographic and clinical characteristics according to serum cholesterol tertiles are presented in Table 1. Gender distribution was equal across the tertiles. The participants in the third tertile were older than in the other tertiles. Higher education was more common in the lowest cholesterol tertile than in the middle and highest tertiles. Employment, living alone, BDI score, smoking, alcohol consumption and physical activity did not show a linear relationship with the serum cholesterol tertiles. The proportions of subjects using medication for blood pressure, diabetes or depression were similar across the tertiles.

**Table 1:** **Baseline characteristics of the study population**

	**Total cholesterol at baseline**			**P for linearity**
	**<4.9**	**4.9–5.7**	**>5.7**	
	**N = 115**	**N = 106**	**N = 105**	
Number of females, %	64 (56)	58 (55)	46 (44)	0.16
Age, years, mean (SD)	47 (10)	51 (9)	53 (9)	<0.001
Education years, mean (SD)	11.5 (3.0)	10.8 (2.8)	10.5 (2.8)	0.026
Living alone, n (%)	11 (10)	9 (9)	16 (15)	0.25
Employed, n (%)	81 (70)	67 (63)	62 (59)	0.20
Medication				
Hypertension	15 (13)	16 (15)	25 (24)	0.084
Diabetes	7 (6)	2 (2)	2 (2)	0.13
Depression	3 (3)	0 (0)	2 (2)	0.27
BDI-21^1^ score, mean(SD)	5.7 (7.3)	4.8 (4.9)	5.8 (6.3)	0.52
Smoking, n (%)	31 (27)	22 (21)	31 (30)	0.32
Alcohol use, n (%)	90 (78)	83 (78)	84 (80)	0.94
Physical activity, n (%)				0.44
Low	22 (19)	17 (16)	15 (14)	
Moderate	36 (32)	25 (24)	32 (31)	
High	55 (49)	64 (60)	57 (55)	

^1^21-Item Beck’s Depression Inventory.

At the baseline, BMI, systolic and diastolic blood pressure, LDL cholesterol level (in females and males), HDL cholesterol level (only in females) and triglyceride levels (in females and males) had a significant linear relationship with the cholesterol tertiles (Table 2). However, HDL cholesterol levels in males, fasting plasma glucose and hs-CRP had no relationship with total cholesterol levels.

**Table 2:** **Baseline clinical and biochemical characteristics and their changes (from baseline to follow-up)**

	**Total cholesterol at baseline**			**p for linearity**
	**< 4.9**	**4.9–5.7**	**> 5.7**	
	**N = 115**	**N = 106**	**N = 105**	
Fasting Plasma cholesterol, mmol/L				
Baseline	4.05	5.28	6.58	-
Change	0.53 (0.36 to 0.71)	-0.29 (-0.43 to -0.14)	-1.23 (-1.46 to -1.01)	0.14*
Body mass index, kg/m^2^				
Baseline	26.2 (5.1)	27.4 (4.4)	28.6 (4.7)	< 0.001
Change	0.59 (0.23 to 0.94)	0.29 (-0.09 to 0.066)	0.03 (-0.34 to 0.41)	0.42*
Fasting Plasma glucose, mmol/L				
Baseline	5.50 (1.14)	5.48 (0.68)	5.47 (0.97)	0.41
Change	0.57 (0.34 to 0.80)	0.33 (0.09 to 0.57)	0.51 (0.27 to 0.75)	0.86*
Fasting Plasma LDL cholesterol, mmol/L				
Males				
Baseline	2.45 (0.55)	3.58 (0.32)	4.45 (0.64)	< 0.001
Change	0.11 (-0.08 to 0.30)	-0.58 (-0.75 to -0.40)	-1.34 (-1.63 to -1.07)	0.47*
Females				
Baseline	2.35 (0.47)	3.10 (0.45)	4.29 (0.66)	< 0.001
Change	0.15 (-0.00 to 0.31)	-0.37 (-0.52 to -0.21)	-1.14 (-1.43 to -0.86)	0.21*
Fasting Plasma HDL cholesterol, mmol/L				
Males				
Baseline	1.05 (0.40)	1.14 (0.29)	1.11 (0.35)	0.45
Change	0.30 (0.22 to 0.37)	0.13 (0.05 to 0.21)	0.16 (0.09 to 0.23)	0.015*
Females				
Baseline	1.27 (0.34)	1.45 (0.41)	1.44 (0.52)	0.026
Change	0.23 (0.15 to 0.31)	0.18 (0.09 to 0.26)	0.17 (0.07 to 0.26)	0.19*
Fasting Plasma triglycerides, mmol/L				
Males				
Baseline	1.12 (0.57)	1.33 (0.67)	1.81 (1.13)	< 0.001
Change	0.07 (-0.18 to 0.31)	0.05 (-0.20 to 0.29)	-0.09 (-0.31 to 0.14)	0.090*
Females				
Baseline	0.88 (0.33)	1.22 (0.60)	1.59 (0.84)	< 0.001
Change	0.14 (0.02 to 0.25)	-0.12 (-0.24 to -0.01)	-0.30 (-0.43 to -0.17)	0.025*
Blood pressure, mmHg				
Systolic				
Baseline	132 (20)	139 (17)	146 (18)	< 0.001
Change	6.1 (3.1 to 9.1)	5.7 (2.6 to 8.9)	6.1 (3.0 to 9.2)	0.050*
Diastolic				
Baseline	79 (11)	3 (10)	87 (11)	<0.001
Change	2.2 (0.35 to 4.0)	1.3 (-0.6 to 3.2)	0.5 (-1.4 to 2.4)	0.036*

Baseline values as means (SD), change values as means with 95% CI.

*Adjusted for age, sex and baseline values.

Table 2 also shows the absolute changes (from the baseline to follow-up) in the aforementioned background variables. There was a linear trend in HDL cholesterol among males—the largest increase was seen in the lowest cholesterol tertile. Respectively, in females, serum triglyceride levels tended to decrease in the higher tertiles. A significant relationship was found between diastolic blood pressure changes and cholesterol tertiles.

The total number of subjects who started to use lipid-lowering medication during the 5 years of follow-up was 64 (19.6%). There was a linear trend between the tertiles. The percentages of subjects with initiated lipid-lowering medication in the first, second and third tertiles were 7.8% (N = 9), 14.1% (N = 15) and 38.1% (N = 40), respectively (age- and sex-adjusted p < 0.001). All lipid lowering drugs were statins, simvastatin (mean dose 30 mg/day) being the most common (81%).

**Figure 2:**
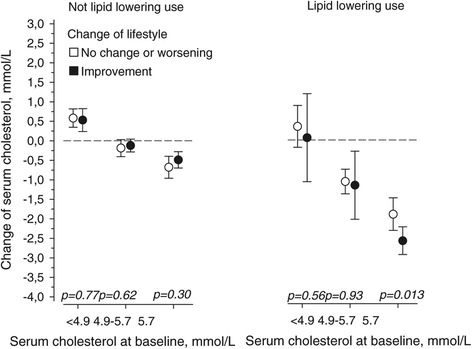
**Change in serum cholesterol level after the five-year follow-up period according to the baseline cholesterol levels in subjects without and with lipid-lowering drugs.**

The change in lifestyle was not associated with baseline cholesterol levels (P = 0.76) or use of lipid-lowering drugs (P = 0.89). Figure 2 presents the mean cholesterol levels in the cholesterol tertiles at the baseline and the change in cholesterol level after five years of follow-up among subjects who had improved their lifestyle and among those who had not. The results are reported separately for the individuals with lipid-lowering medication and for those without. The difference between those who had improved their lifestyle and those who did not improve was significant only among subjects with medication in the highest baseline cholesterol tertile. The cholesterol levels decreased among the subjects in the highest tertile without lipid-lowering medication. Respectively, cholesterol level decreased among the subjects who used medication in the second and highest tertiles. In general, the change in cholesterol level was associated with medication (P < 0.001). Lifestyle improvement was associated with the cholesterol level change, but did not quite reach statistical significance (P = 0.061). However, the interaction of lipid-lowering medication and lifestyle change was associated with the cholesterol level change (P = 0.018). Compared with total cholesterol, a corresponding change was found in the LDL cholesterol change. However, the corresponding findings for HDL cholesterol and triglyceride changes were not significant.

A regression analysis included age, sex, baseline cholesterol, the particular components of lifestyle changes (smoking, use of alcohol, physical activity, use of salt, use of vegetables, fruits or berries and use of fat in meals) and lipid-lowering medication (Table 3). In a univariate analysis age, baseline cholesterol level, lipid lowering medication and the favourable change in the use of fat in meals were associated with the favourable change of cholesterol level. In a multivariate forward stepwise method, baseline cholesterol level, lipid lowering medication and change in the use of fat in meals entered the model.

**Table 3:** **Association of age, gender, baseline cholesterol values and particular lifestyle items with the change of cholesterol level**

	**Univariate**		**Multivariate** ^**1**^	
**Variables**	**β** ^**2**^ **(95% CI)**	**P-value**	**β** ^**2**^ **(95% CI)**	**P-value**
Gender (Male)	−0.12 (−0.26 to 0.01)	0.067		
Age, years	−0.27 (−0.40 to −0.14)	<0.001		
Total cholesterol at baseline, mmol/l	−0.86 (−0.96 to −0.77)	<0.001	−0.59 (−0.66 to −0.52)	<0.001
Lipid-lowering medication	−0.56 (−0.65 to −0.47)	<0.001	−0.37 (−0.44 to −0.30)	<0.001
Change of life style item:				
Smoking	0.05 (−0.09 to 0.18)	0.48		
Alcohol	−0.08 (−0.21 to 0.06)	0.26		
Exercise	0.06 (−0.07 to 0.20)	0.35		
Berries and fruits	0.10 (−0.03 to 0.23)	0.14		
Salt	0.01 (−0.13 to 0.14)	0.97		
Use of fat in meals^3^	−0.19 (−0.32 to −0.06)	0.006	−0.09 (−0.16 to −0.03)	0.007

^1^Forward selection. Only those variables are shown that entered (significance level for addition to the model was p < 0.05) the model.

^2^Standardized regression coefficients.

^3^Combined fats in meals, spreads and drinks.

## Discussion

The present community and primary care setting findings suggest that, at the population level, individual cardiovascular risk factor levels can be improved with lifestyle changes and use of drugs. A lowering of cholesterol levels was achieved significantly among those people who used statins, as expected, although a favourable change in lifestyle had at least an additive influence on serum cholesterol level.

Statins are highly effective in lowering serum cholesterol concentration and preventing ischemic heart disease [22-24]. In dietary intervention studies, plasma lipid and lipoprotein responses have been variable [25-29]. Yu-Poth’s study shows that individuals with marked elevations in total cholesterol and LDL cholesterol were less responsive to dietary interventions than mildly to moderately hypercholesterolemic individuals. Supporting those findings, our study indicated that higher cholesterol levels were not associated with more significant lifestyle changes than lower levels in general [30]. Lytsy et al. showed that patients taking statins seemed to have a healthier lifestyle than non-statin users [11]. The present study did not find this kind of trend. However, the present findings suggest that favourable lifestyle changes are related to decreasing cholesterol levels among statin users. It is possible that need for daily chronic medication by some mechanisms activates patients to make lifestyle changes.

The present study was based on a sample from one community in a catchment of one primary health care centre. In a community with active primary care, the most significant factor behind a decreasing level of cholesterol may be the use of cholesterol-lowering drugs. However, the favourable change in fat consumption found in the present study seemed to also play a role in this community. It is probable that the potential of influencing the health behaviour of individuals and the population in general is not used as effectively as it could be [31]. Prescribing statins may be quite an easy intervention for a general practitioner (GP). On the other hand, attempts to influence lifestyle and promote health can be more difficult, although counselling delivered by the GP can be effective [32]. In addition to intrapersonal factors, recognised barriers to health promotion may include interpersonal, institutional or community factors [31]. The traditional biomedical model adopted by many physicians may result in treatment rather than prevention. They may perceive a lack of health promotion training and evidence about the effectiveness of health-promoting actions [31]. In the present study, cholesterol level changes were desirable and related most strongly to lipid-lowering medication. Contrary to these, BMI, plasma glucose and blood pressure changes were not desirable. A trend towards positive lifestyle changes seemed to have no impact on other measures except lipids. The present results support the view that, in addition to individual medical treatment, population-based strategies have been sub-optimally adopted in communities [33].

The study area—Lapinlahti municipality—represents a semirural community. Therefore, these results may not be directly generalised to more urban populations. However, primary health care in Finland has better population coverage in rural and semirural areas than in urban areas [34]. Hence, these results represent communities with strong primary health care in which GPs have significant potential to influence treatment and health behaviour. The information about nutrition components of lifestyle was based on the structured questions but not on a food diary. Therefore, we could assess general eating patterns but not intake of various food items. The investigation focused on key risk factors and their changes. The present study represents the general population in one community. The measurements were conducted in a similar way at the baseline and follow-up. Likewise, lifestyle was measured in the same way at both examinations. We did not have access to patient records. Therefore we do not know the individual cholesterol levels at the time point of starting lipid lowering medication. In general, the reasons for the initiation of treatment in the Finnish primary health care followed the national guidelines [35].

The impact of medication was obvious, because it was started during the follow-up and evidently was in line with the observed changes in serum cholesterol.

## Conclusions

At the population level, dyslipidaemia is one of the most easily modifiable risk factors of CHD. This study indicates that lipid-lowering medication may have the most significant impact on cholesterol level in communities with primary health care with good coverage. On the other hand, the potential of health-promoting and population-based prevention strategies may be underused.

## Abbreviations

ANOVA: Analysis of variance
BDI: Beck´s depression inventory
BMI: Body mass index
CHD: Coronary heart disease
HDL: High density lipoprotein
LDL: Low density lipoprotein
